# Graft survival after percutaneous transluminal renal stenting for transplant renal artery stenosis (TRAS) is worse compared to matched cadaveric grafts without TRAS

**DOI:** 10.1080/0886022X.2024.2378211

**Published:** 2024-07-31

**Authors:** Long Zhang, Jilin Zou, Jiangqiao Zhou, Tao Qiu, Chenyang Kong, Tianyu Wang, Zhongbao Chen, Xiuheng Liu

**Affiliations:** a Department of Organ Transplantation, The People’s Hospital Affiliated Wuhan University, Wuhan, China; b Department of Urinary Surgery, The People’s Hospital Affiliated Wuhan University, Wuhan, China

**Keywords:** Transplant renal artery stenosis, kidney transplant, stent placement, interventional therapy, graft survival

## Abstract

**Objectives:**

Transplant renal artery stenosis (TRAS) is now recognized as a curable disease with a good prognosis if intervention occurs in the early stage. However, the mid-term outcomes of TRAS when treated by percutaneous transluminal angioplasty with stent placement have yet to be fully elucidated. The purpose of this study was to compare mid-term graft and patient survival of TRAS group with a control group.

**Patients and methods:**

Ninety-two patients were diagnosed of TRAS between January 2016 and January 2022 in our center. Fifty-six pairs of recipients with grafts from the same donor were selected as a study group with TRAS and a control group without TRAS, respectively. All donor kidneys were from deceased organ donation rather than living donors. The primary endpoints were graft and patient survival. The secondary outcomes were changes in renal graft function.

**Results:**

The mean follow-up time for the TRAS group was 43.6 months, while the mean follow-up time for the control group was 45.3 months. In the TRAS group, the age of patients ranged from 11 to 62 years with 39 males and 17 females. In the control group, the age of patients ranged from 18 to 67 years with 40 males and 16 females. In the TRAS group, there were more patients with diabetic nephropathy as the primary renal disease compared to the control group (5/56 vs 0/56), and the incidence of acute rejection was higher in the TRAS group than in the control group (12/56 vs 3/56). Eight patients in the TRAS group and one patient in the control group experienced graft loss (*p* = .019). Four patients in the TRAS group and four patients in the control group died with functional renal allograft during the follow-up time (*p* = .989). The levels of eGFR did not differ significantly between the two groups in the first three years after kidney transplant (*p* > .05). Patients in the TRAS group had worse graft functionality (eGFR, 44.96 ± 18.9 vs 54.9 ± 19.6 mL/min) in the fourth year when compared with the control group (*p* = .01).

**Conclusions:**

The graft function deteriorated faster, and graft survival was lower in the TRAS group treated by stent placement when compared with a control group without TRAS over the mid-term.

## 1. Introduction

The number of kidney transplantations has increased rapidly along with the initiation of the donation after citizens’ death (DCD) policy in China since 2012. An increasing number of renal allografts with arterial atherosclerosis from older cadaveric donors have been used over the last decade. It is well-known that transplant renal artery stenosis (TRAS) is more likely to occur in grafts with arterial atherosclerosis [1]. Color Doppler ultrasound or contrast-enhanced ultrasound is widely used for screening TRAS, while CT angiography (CTA) or magnetic resonance angiography (MRA) is employed for further evaluation when ultrasound is inconclusive for diagnosing TRAS. This condition usually occurs between 3 months and 2 years after kidney transplantation but can present at any time depending on different etiologies [2]. TRAS may arise early from unexpected injury of the renal transplant artery during surgery or later from atherosclerotic disease [3]. The prevalence of TRAS ranges between 1% and 23% depending on the definition and diagnostic techniques employed. The clinical presentations are worsening or uncontrolled hypertension and/or exacerbated graft function. If timely and effective treatments are not taken, TRAS may cause graft loss and even premature death of the recipient.

Conservative therapy, angioplasty and surgery are the main treatments for TRAS. Conservative therapy can be indicated when graft function is stable, and there is no hemodynamically significant stenosis, as determined by different screening methods [4]. Percutaneous transluminal renal angioplasty (PTA)/stenting can restore graft kidney perfusion in 70%–90% cases [5]. PTA alone may increase the risk of restenosis in 10%–30% cases over a period of 6–8 months [6]. Stent placement at the first PTA procedure may reduce the incidence of TRAS recurrence [7]. Surgery can be performed for patients with ostial stenosis associated with external iliac artery stenosis or early bifurcation, and kinking for which PTA is unsuitable or for those who have failed an initial PTA [8].

Although many studies have reported the outcomes of different treatments for TRAS, rigorous control groups were scarce; in particular, previous studies tended to ignore the influences of donor grafts. It is well known that the quality of a renal graft plays a crucial role on long-term patient and graft survival. As a result, there many controversial or contradictory results have been reported with regards to the therapeutic effects of TRAS. To avoid the interference from donor kidneys, our study selected recipients without TRAS who received allografts from the same donor as the control group. The primary objective of this study was to explore early- and mid-term graft and patient survival between the TRAS and control groups. The secondary objective was to investigate trends in serum creatinine and proteinuria during the follow-up period.

## 2. Patients and methods

The study protocol was approved by the Medical Research Ethics Committee of our hospital and the approval code for this study was WDRY2022-K181. We committed to protecting the privacy of patients during the collection of clinical data. The protocol conformed to the ethical guidelines of the 1975 Helsinki Declaration, and we, hereby, assure that all of the kidney transplant surgeries performed in our center were in compliance with the Istanbul Declaration regarding organ trade and transplant tourism. We also have obtained the written informed consent from patients or their guardians. In the process of allocating donor kidneys, the China organ transplant response system matched donors and recipients based on their BMI and ensure that the BMI of both are similar.

### 2.1. Selection of the study and control group

**Figure 1.**
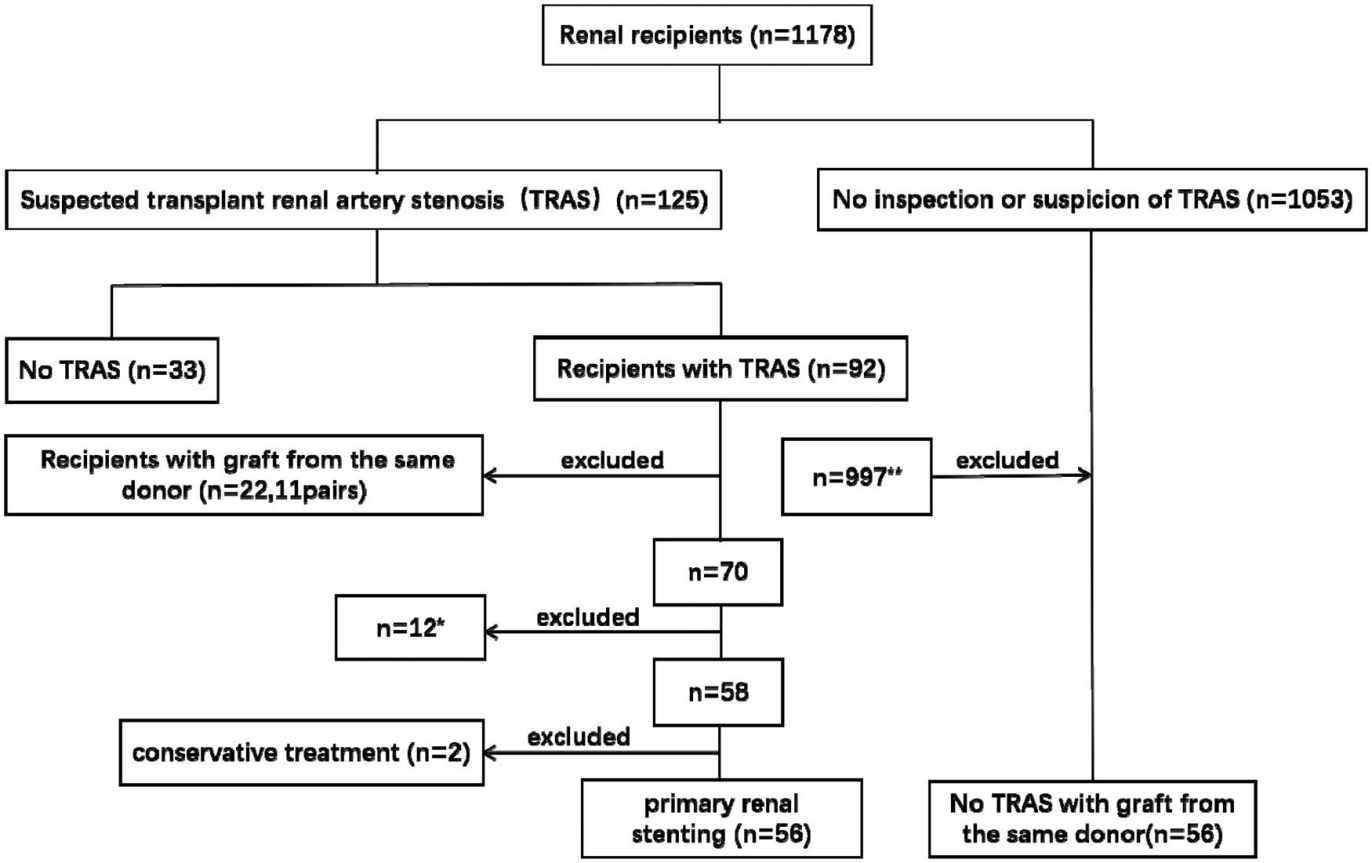
Flow chart showing the recruitment process for two groups (the TRAS group and the matched control group without TRAS). *The 12 recipients were excluded from study group as the other 12 recipients with grafts from the same donor were lost to follow-up. **These 997 patients were excluded from the control group as their grafts originated from other donors.

Between January 2016 and January 2022, 1178 patients underwent renal transplant in the People’s Hospital Affiliated Wuhan University. The inclusion criteria were as follows: (1) Paired recipients were selected as study group and control group with donor kidneys from the same donor; (2) The study group had TRAS, while the control group did not have TRAS. Patients were excluded if they met any of the subsequent criteria: (1) had undergone relative kidney transplantation; (2) had undergone multiple kidney transplantations or multiorgan transplantation; (3) possessed incomplete clinical data. TRAS was not identified in 1053 cases of patients and 125 cases of patients suspected of having TRAS. Of these, 92 were diagnosed with TRAS by noninvasive or invasive procedures, while 33 cases of patients were diagnosed without TRAS. Eleven pairs (22) of recipients with grafts from the same donor were diagnosed with TRAS at different time points. Twelve recipients were excluded from study group as the other 12 recipients with grafts from the same donor were lost to follow-up. Of the remaining 58 recipients, two patients had no treatment, 56 patients had stent placement. Because the renal graft functions of these two patients are stable, hypertension can be effectively controlled through antihypertensive medication. Through MRA and interventional angiography, we found that the renal artery stenosis in these two recipients was mild to moderate. Therefore, both patients opted for conservative treatment. We chose the other 56 recipients with grafts from the same donor who did not have TRAS as a control group (Figure 1). Kidney transplant surgeries (end-to-side anastomosis) were all performed by our team. Typically, paired recipients undergo surgery concurrently in two separate operating rooms with two surgical teams, resulting in similar cold ischemia times.

### 2.2. Diagnosis of TRAS and interventions

We regularly followed up all patients who received kidney transplants in our department weekly for the first 3 months, half-monthly from 3 months to 1 year, and then every month after the first year. Both groups of patients regularly undergo blood and urine routine tests, liver and kidney function tests, and monitoring of immunosuppressive agent concentration at our outpatient clinic. Both groups of patients adhere to medication schedules and follow-up appointments regularly, demonstrating good compliance.

Each patient who had unexplained graft dysfunction or uncontrolled hypertension would first receive Doppler ultrasound screening. MRA/spiral CTA were further used if the velocity of the transplant renal artery exceeded 200 cm/s by Doppler ultrasound screening. Arteriography was the last step for the diagnosis and treatment of TRAS. Asymptomatic cases with a clinical concern of TRAS by noninvasive procedures also underwent arteriography.

Transplant renal arteriography was performed by interventional radiologists via femoral artery puncture on a regular basis. We relied on luminal diameter reduction as the major diagnostic indicator of TRAS. Patients with a reduction of luminal diameter by >50% were treated by PTA/stent placement. We measured the normal part of the renal transplant artery to confirm the diameter of the stent and balloon. Each patient was followed-up by our team after their procedure, and any immediate complications were noted. Clopidogrel was routinely used on a daily basis for 6 months after PTA with stent placement.

### 2.3. Data collection and study end points

Data was collected from January 2016 to January 2023 (by LZ). We retrieved demographic data and the outcomes of all enrolled patients from our department’s electronic patient record system. We assessed graft and patient outcomes through follow-up in the outpatient department. Graft function outcomes were assessed by testing the serum levels of creatinine, urea nitrogen and eGFR.

Graft survival and patient survival were the first end points. Graft loss was defined as a return to dialysis. Trends in graft function were the secondary end points.

### 2.4. Statistical methods

We used the Student’s *t*-test and Fisher’s exact test to compare continuous variables after normality tests. We also used Kaplan–Meier plots, Cox-proportional-hazards regression tests and log-rank tests to investigate graft survival and patient survival. All statistical analyses were conducted with SPSS (IBM Corporation, Armonk, NY, USA) or GraphPad Prism 8.0.2 (GraphPad Software, San Diego, CA, USA), and *p* < .05 was considered statistically significance.

## 3. Results

**Figure 2.**
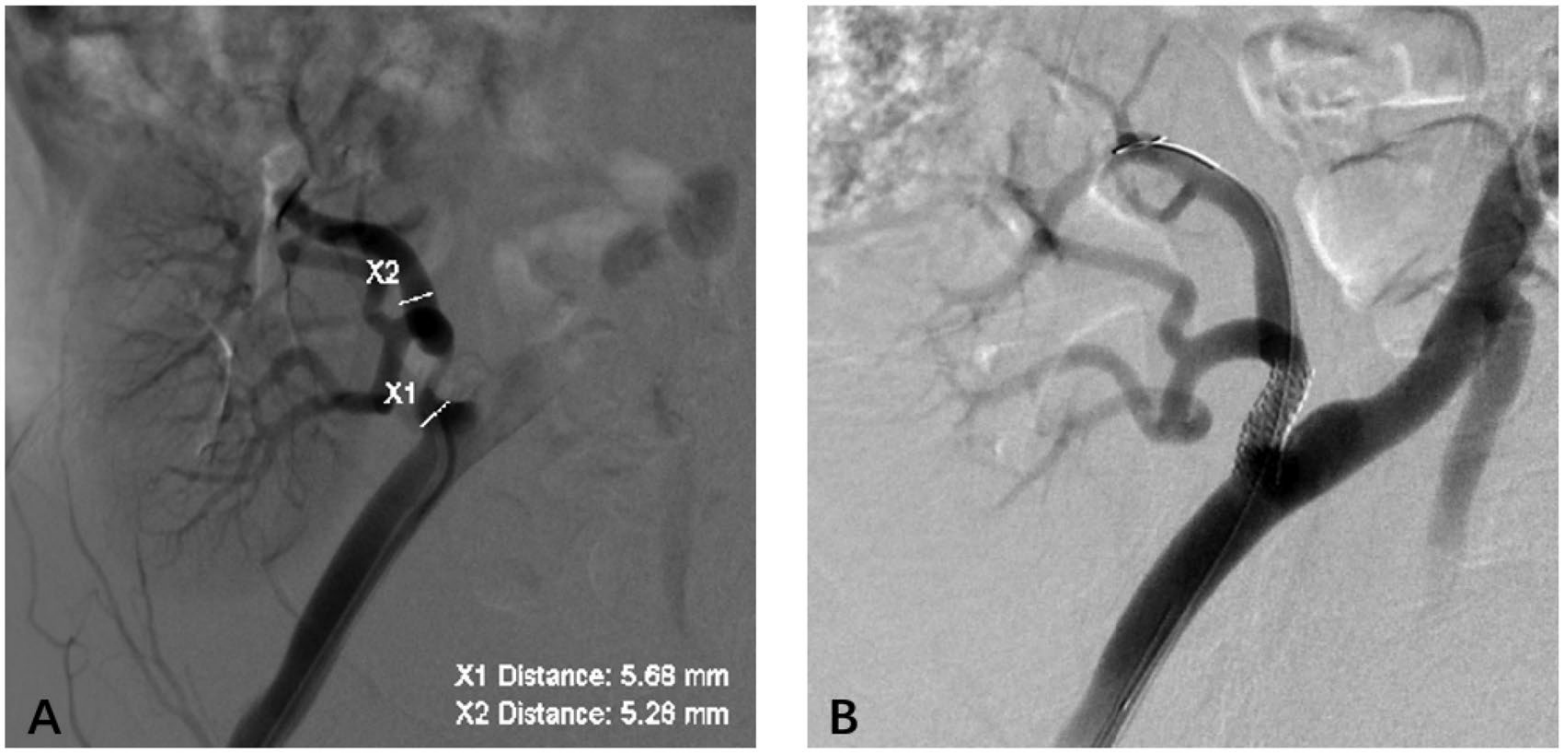
The treatment of TRAS by PTA with stent placement. (A) Preoperative angiography revealed the location of stenosis as the renal transplant artery. We measured the normal diameter of the artery. (B) We treated TRAS by PTA with stent placement and restored normal blood supply to the graft. TRAS: transplant renal artery stenosis; PTA: percutaneous transluminal renal angioplasty.

In total, 1178 patients received kidney transplantations (KT) with 37 living renal grafts and 1141 cadaveric grafts between January 2016 and January 2022 in our center. One hundred and twenty-five patients were suspected to have TRAS and underwent further inspections (MRA/CTA/arteriography). Thirty-three patients were excluded from the study group after further examination confirmed the absence of TRAS. Of these, 92 cases were diagnosed with TRAS and were treated by different methods. All the 92 patients who were diagnosed with TRAS received cadaveric grafts. Three patients without any treatments experienced thrombosis of the transplant renal artery and lost their grafts. Three patients underwent balloon angioplasty. Eighty-six patients underwent stent placement, as shown in Figure 2. The follow-up period ranged from 15 months to 80 months. The diagnosis of TRAS was confirmed 19 days to 36 months after kidney transplantation (median: 8.4 months).

### 3.1. Comparison of basic demographics between TRAS and matched control groups

There were no statistically significant differences in age, sex, the cause of end stage renal disease (ESRD), the prevalence of diabetes, delayed graft function (DGF), or serum lipids level between the TRAS and matched control groups (Table 1). Induction therapy was performed for all recipients. The most commonly used induction agent was Basiliximab. Tacrolimus combined with mycophenolate and prednisolone was the most commonly used maintenance immunosuppressant regimen. We routinely maintain FK506 concentrations at 8–10 ng/ml in the first year post-surgery, 6–8 ng/ml in the 2–5 years post-surgery, and 5–7 ng/ml in the 6–10 years post-surgery.The use of induction therapy and maintenance immunosuppressant regimen was similar between the study and control groups (*p* > .05). In addition, the incidence of acute rejection was significantly higher in the TRAS group than in the control group (*p* = .02). The mean peak renal artery velocity in the TRAS group was 314.6 cm/s above the normal level and was significantly higher than in the control group (*p* < .001).

**Table 1.** Demographic and clinical data of patients with TRAS and a matched control group without TRAS from the same donor.

Characteristics	TRAS group (*n* = 56)	Control group (*n* = 56)	*p*-value^ζ^
Sex			.84
Male	39	40	
Female	17	16	
Median age (y)^α^	42 (11–62)	43 (18–67)	.38
Male patients	42 (11–62)	42 (18–67)	
Female patients	44 (23–56)	44 (22–55)	
Cause of ESRD^β^			>.05
Glomerulonephritis	45 (80.3)	43 (76.8)	
Gouty nephropathy	0	4 (7.1)	
Diabetic nephropathy	5 (8.9)	0	
PKD	1 (1.8)	1 (1.8)	
IgAN	2 (3.6)	7 (12.5)	
Nephrolithiasis	2 (3.6)	1 (1.8)	
Others	1 (1.8)	0	
Medical history			
DGF^γ^	13	12	.82
Diabetes	8	3	.20
Hypertension	56	53	.24
History of rejection^δ^	12	3	.02*
Immunosuppression^β^			>.99
Tacrolimus	54 (96.4)	53 (94.6)	
Mycophenolate mofetil	56(100)	56 (100)	
Cyclosporine	2 (3.6)	3 (5.4)	
Sirolimus	1 (1.8)	1 (1.8)	
Prednisolone	54 (96.4)	55(98.2)	
Mizoribine	1 (1.8)	0	
Induction			>.99
Basiliximab	54	53	
ATG	2	3	
Serum lipids (mmol/l)^ε^			
Triglyceride	1.82 ± 1.01	1.76 ± 1.18	.77
Total cholesterol	4.20 ± 1.01	4.15 ± 1.22	.85
PRAV (cm/s)^ε^	314.6 ± 128.1	142.4 ± 81.5	<.05*

ESRD: end stage renal disease; PKD: polycystic kidney; IgAN: IgA nephropathy; DGF: delayed graft function; ATG: antithymocyte globulin; SD: standard deviation; PRAV: peak renal artery velocity.

^α^Data in parentheses represent age range at transplantation.

^β^Data in parentheses represent percentages.

^γ^DGF was defined when recipients required post-operative dialysis in the first week after kidney transplantation.

^δ^Rejection occurred at least once and was diagnosed by graft biopsy or clinical judgment.

^ε^Data represent the mean and standard deviation of serum lipids and peak renal artery velocity.

^ζ^*T*-test or Fisher’s exact test were used as appropriate.

*p < .05 was considered statistically significant.

### 3.2. Trends in the graft function of the TRAS group after stent placement

**Figure 3.**
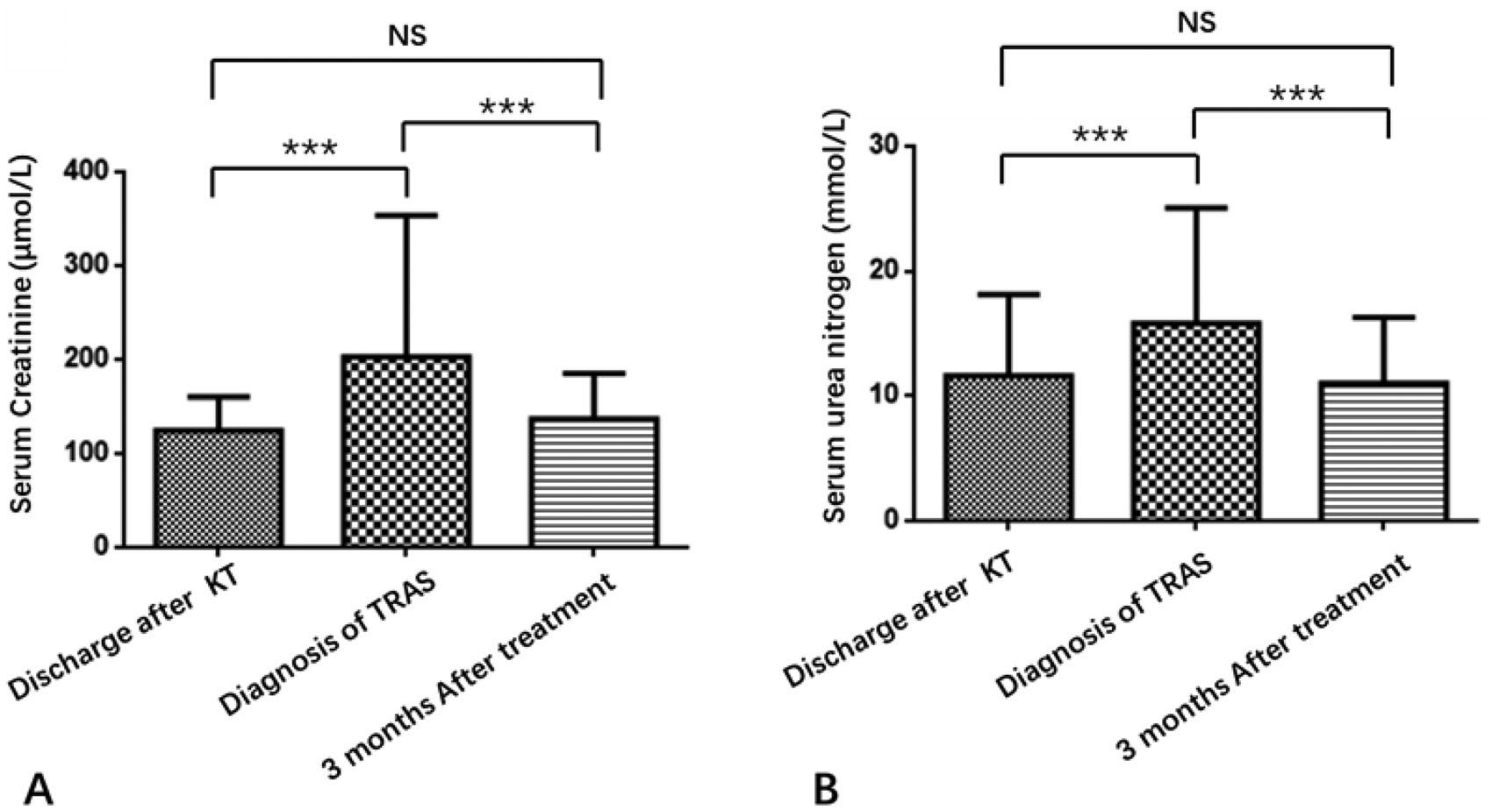
Trends of serum creatinine and urea levels at three different time points. ***p < .001; NS: no significance.

We compared the sCr and BUN at three different time points: the time of discharge after transplantation, the time of TRAS diagnosis, and 3 months after stent placement for TRAS. It is well known that contrast media used during interventional therapy may cause acute graft injury. We found that the levels of sCr and BUN did not change significantly over the short-term after interventional therapy. To eliminate the potential influence of contrast media, we chose sCr and BUN levels at 3 months after interventional therapy to evaluate the curative effect. As shown in Figure 3, the levels of sCr and BUN increased significantly when TRAS occurred and decreased rapidly 3 months after stent placement (*p* < .001). There was no statistical difference with respect to sCr and BUN levels between the time of discharge after transplantation and 3 months after stent placement.

### 3.3. Comparison of outcomes between the TRAS and matched control groups

**Figure 4.**
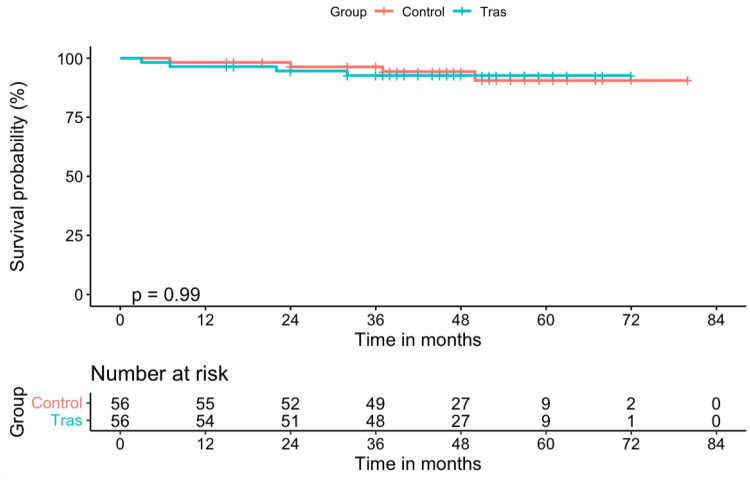
Patient survival for 56 recipients with TRAS after stent placement, as compared with the matched control group. There was no statistical difference in patient survival between the two groups (*p* = .989, log-rank test).

**Figure 5.**
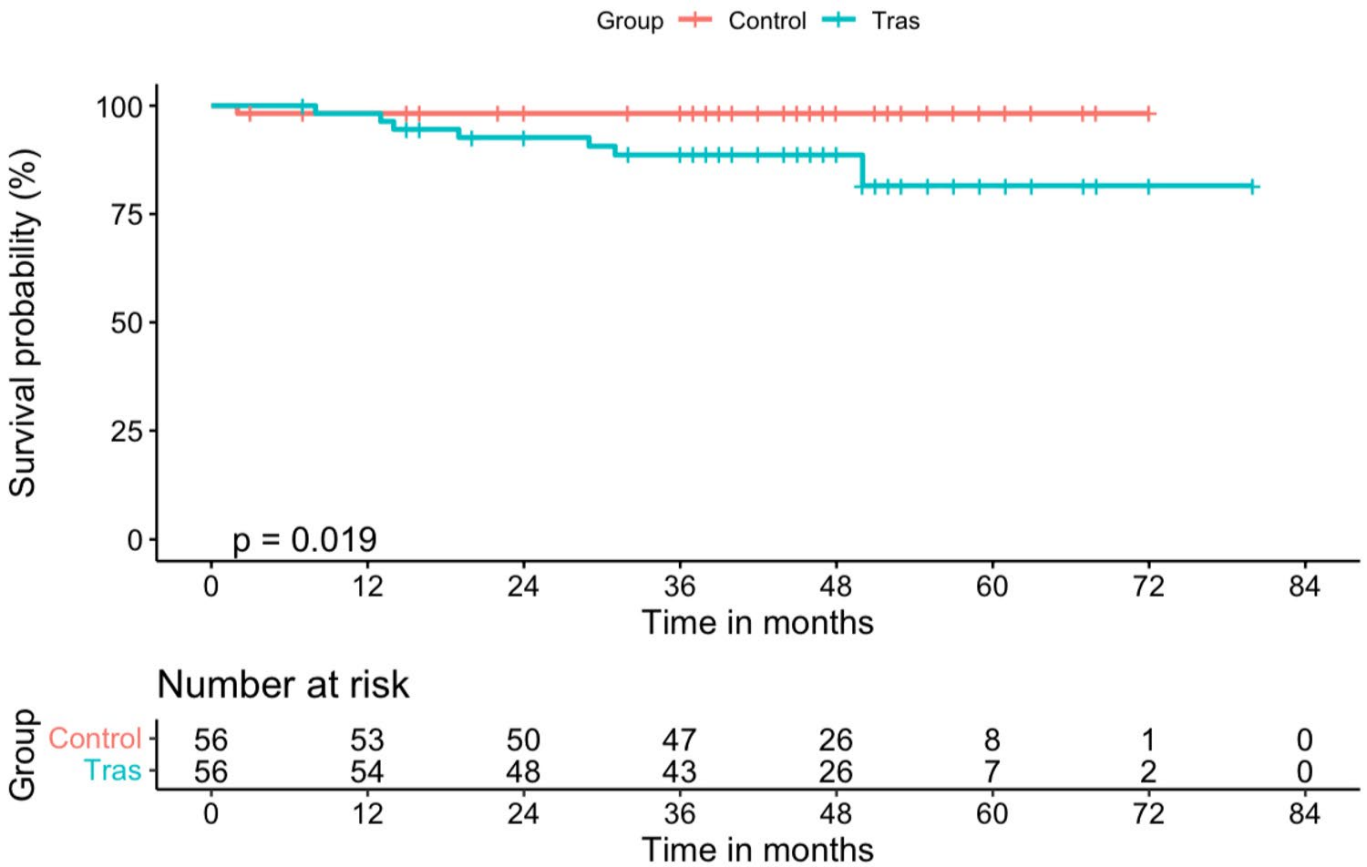
Graft survival of 56 recipients with TRAS after stent placement, as compared with the matched control group. The incidence of death censored graft failure was significantly higher in the TRAS group (*p* = .019, log-rank test).

During the follow-up period, 8 of the 112 patients died (4 patients in the TRAS group and 4 in the control group), as shown in Table 2. There was no statistical difference in patient survival between the two groups (*p* = .989), as shown in Figure 4. Three patients died of pulmonary infections and one patient died of aortic dissection in the TRAS group. Two patients died of pulmonary infections, one died of heart failure, and one died of unknown causes in the control group. However, the incidence of death-censored graft failure was significantly higher in the TRAS group (*p* = .019) given that eight patients in the TRAS group suffered death‐censored graft failure during the study period, as shown in Figure 5. One patient experienced digestive tract hemorrhage and premature graft loss by hemorrhagic shock in the control group. The causes of graft failure and patient death are summarized in Table 3. In the TRAS group, the most common causes of graft failure were chronic allograft nephropathy and the recurrence of nephropathy. Seven patients returned to hemodialysis and one patient received a second kidney transplant. In the control group, the only one patient who lost his graft returned to hemodialysis.

**Table 2.** Comparison of outcomes between the TRAS and matched control group.

Variables	TRAS group (*n* = 56)	Control group (*n* = 56)	*p* value
Patient deaths	4	4	.989
Death‐censored graft failure	8	1	.019*

*p < .05 was considered statistically significant.

**Table 3.** The etiology of graft losses and deaths in the two groups.

Variables	TRAS group	Control group
Graft loss	8	1
Recurrence of nephropathy	3	0
Acute rejection	1	0
Chronic allograft nephropathy	4	0
Hemorrhagic shock	0	1
Outcomes after graft failure		
Second transplant and alive at last follow-up	1	0
Alive on hemodialysis	7	1
Patient deaths	4	4
Aortic dissection	1	0
Pulmonary infection	3	2
Heart failure	0	1
Unknown	0	1

### 3.4. Trends in graft function at different time points in the two groups

The levels of eGFR and proteinuria were compared between the two groups at different time points after kidney transplant (Tables 4 and 5). The mean and median eGFR levels were higher in the TRAS group when compared with the control group in the first 3 years after kidney transplantation; however, these differences were not statistically significant (*p* > .05). In the fourth year after kidney transplantation, the mean and median level of eGFR was significantly higher in the TRAS group than in the control group (*p* = .01). We can find from Table 4 that the eGFR levels in TRAS group broadly, slowly deteriorated whilst they stayed steady in the control group at the over 4 years. A similar situation occurred with regard to the number of patients who developed proteinuria. The number of patients who developed proteinuria was not statistically significant when compared between the two groups in the first 3 years (*p* > .05). However, a significantly greater number of patients in the TRAS group developed proteinuria in the fourth year (*p* = .04).

**Table 4.** Trends in eGFR after kidney transplantation in the two groups.

Parameter^α^	TRAS group					Control group					*p* ^β^
	NO. of patients	eGFR (mL/min)				NO. of patients	eGFR (mL/min)				
		Mean	Median	95%CI	SD		Mean	Median	95%CI	SD	
3 months	56	64.1	65.7	58.4-69.8	21.2	55	66.3	65.5	61.1-71.6	19.6	.57
6 months	56	60.7	60.2	55.8-65.7	18.6	54	64.5	65.4	59.1-69.9	20.0	.31
1 year	54	58.3	60.0	52.8-63.7	20.2	53	59.3	60.1	53.5-64.9	20.5	.79
2 years	48	54.1	55.5	48.5-59.7	20.4	51	57.0	55.0	51.8-62.3	19.1	.44
3 years	45	50.9	51.9	45.6-56.3	19.5	48	56.4	52	50.9-61.9	19.7	.16
4 years	32	44.96	49.0	39.5-50.4	18.9	35	54.9	52.8	49.3-60.6	19.6	.01*

^α^eGFR were measured at different times after kidney transplantation.

^β^The Mann–Whitney test was used as data did not comply with the normal distribution.

*p < .05 was considered statistically significant.

**Table 5.** Trends in proteinuria after kidney transplantation in the two groups.

Parameter	TRAS group						Control group						*p*
	No. of patients	Urine protein					No. of patients	Urine protein					
		Negative	Trace	1+	2+	3+		negative	Trace	1+	2+	3+	
3 months	56	44	7	5	0	0	55	43	6	4	1	1	.96
6 months	56	42	9	3	2	0	54	40	0	11	2	1	.91
1 year	54	37	10	5	2	0	53	39	5	6	3	0	.56
2 years	48	36	4	5	3	0	51	39	4	4	4	0	.87
3 years	45	30	4	7	2	2	48	32	4	8	3	1	1
4 years	32	16	8	6	1	1	35	26	2	6	1	0	.04

### 3.5. The outcomes of paired patients (grafts from the same donor) with TRAS

Eleven pairs (22 patients) who received grafts from the same donor were diagnosed with TRAS. Of the 22 patients, stent placement was performed for 19 patients, balloon angioplasty was performed for two patients, and one patient was not treated (Supplementary Table 1). Of the 19 patients with stent placement, 5 patients (26%) had a normal level of sCr, 8 patients (42%) developed graft dysfunction, 2 patients (11%) lost graft function, and 4 patients (21%) died with functioning grafts. Two patients lost grafts caused by chronic allograft nephropathy and returned to hemodialysis. Three patients died of pulmonary infection and one patient died of tuberculous meningitis. Balloon angioplasty was performed for two patients, one had normal levels of sCr and one patient lost his graft due to chronic allograft nephropathy. One patient who did not receive any treatment lost his graft due to renal transplant artery thrombosis and received a second renal transplantation.

### 3.6. Post procedure complications of TRAS group

There were three post procedure complications in 56 patients from the TRAS group, resulting in an overall incidence rate of 5.36%. One patient developed a pseudoaneurysm at the femoral artery puncture site, which was successfully treated by applying pressure to the puncture site with a compressor, leading to no further enlargement of the aneurysm and thrombus formation in the cavity. The patient’s creatinine level was 82 μmol/L after a 4-year follow-up. Another patient had a hematoma at the puncture site, which stopped bleeding after compression and had a creatinine level of 214 μmol/L after a 6-year follow-up. One patient developed a subcapsular hematoma in the transplant kidney caused by guidewire puncture, which was treated with pressure bandaging and blood transfusion, maintaining stable graft function. However, the patient passed away two months later due to disseminated fungal infection.

## 4. Discussion

The prevalence of identified TRAS in our center was 8.7%; this was consistent with previously published studies. Our study demonstrated that stent placement for TRAS can achieve good curative effects in the short term, as reported in previous studies [9,10]. However, a comparative study reported by Wong et al. showed that patient and graft survival were both lower in a TRAS group compared with a control group over 6.9 years of follow-up [11]. In contrast, a 21-year matched cohort study reported that the long-term patient and graft survival of TRAS when treated by PTA did not differ significantly when compared to recipients without TRAS [12]. Our present study showed that the sCr level of patients with TRAS were significantly higher in the fourth year after kidney transplantation. In addition, we observed that eight patients in the TRAS group lost their grafts while only one patient in the control group lost his graft prematurely due to digestive tract hemorrhage. Over time, recipients may suffer ischemic nephropathy due to TRAS caused by various reasons, thus leading to irreversible renal parenchymal damage [2,13,14]. Prolonged graft kidney ischemia may cause irreversible ischemic changes; as a result, graft function cannot recover fully even after restoring effective blood flow [15]. As previously reported, an increased resistance index, as determined by Color Doppler ultrasound, may reflect that a renal graft may have developed structural changes and cannot fully recover after revascularization [16].

Our study involved paired recipients with grafts from the same donor as a study group with TRAS and a control group without TRAS. This ensured the similar quality of donor grafts between the TRAS and control groups. We guess that the small sample size and selection bias of the study and control groups in previous studies led to contradictory findings. We believe that our present sample size of TRAS patients and our more rigorous controlled group could increase the reliability of our study.

Of note, all patients who received living renal allografts did not experienced TRAS. It is possible that the shorter cold ischemia time and fewer immunological factors associated with live-donor renal transplantation may have reduced vascular damage and fibrosis [17]. Usually, TRAS arising several years after kidney transplant may be associated with atherosclerotic disease. The presence of atherosclerotic plaques in transplant renal artery or Carrel patch is more related to the occurrence of TRAS. Therefore, when atherosclerotic plaques are present in the Carrel patch, we usually remove the patch, which helps reduce the occurrence of TRAS. Furthermore, timely administration of lipid-lowering medication to recipients with elevated blood lipid levels may be advantageous in stabilizing atherosclerotic plaques, thus reducing the occurrence of long-term postoperative TRAS. In our center, most TRAS cases developed several months after kidney transplantation. We suggest that the renal transplant artery intima may have been injured during surgery. Furthermore, a few marginally appropriate renal grafts were used in our center due to organ shortage. These marginal appropriate grafts may have poor vascular condition, thus leading to the premature occurrence of TRAS.

We also found that more recipients in the TRAS group had a history of acute rejection when compared with the control group. As described previously, endothelial activation caused by acute rejection may upregulate the expression of ICAM-1 and VCAM-1, eventually leading to microvascular and macrovascular damage [18]. The presence of diffuse stenosis can indicate immune-mediated endothelial injury, as evidenced by the histological similarities observed between the narrowed arteries and the vessels affected by renal allograft rejection. [19,20]. Additionally, the development of post-anastomotic TRAS has been proven to be relevant with *de novo* Class II donor-specific antibodies [21].

In our results, three patients, who did not receive treatments, developed thrombosis of the renal transplant artery, which eventually caused graft loss. Complications such as vomiting, diarrhea, and COVID-19 infection can cause hemodynamic changes or coagulation disorders; under such conditions, patients with TRAS are more likely to develop kidney allograft infarction [22]. We suggest that the degree of stenosis must be carefully evaluated by multiple screening methods and we recommend that PTA with or without stent placement should be performed in time when narrowing of the transplant renal artery exceeds 50% to prevent unexpected thrombosis. Considering the medical burden and risk of complications, we should be very considerate when carrying out PTA.

Eleven pairs of recipients with grafts from the same donor developed TRAS during the follow-up time. These grafts have poorer vascular conditions and suffer longer cold ischemia times. The longer time of cold ischemia superimposed with reperfusion may lead to the marked production of oxygen free radicals and cause damage to endothelial cells, blood vessels and the parenchyma [23,24]. This led to premature development of TRAS in these patients, with a majority experiencing a poor prognosis.

In addition, we only analyzed the medium-term outcomes of stent placement for TRAS, thus avoiding the bias from different treatments. Balloon angioplasty was performed in only three patients; we do not discuss the therapeutic effects of this procedure in detail as this was not the primary focus of the current study. As with most other studies, our research was performed in a single center and there may have been bias from operators and readers during the screening and treatments deployed for TRAS. Furthermore, we did not fully analyze blood pressure due to multiple interference from blood pressure medications. In addition, the mechanism of proteinuria and the aggravation of graft function in the TRAS group is worthy of further research.

In conclusion, short-term graft and patient survival following stent placement for TRAS were similar to that of recipients without TRAS. However, graft function deteriorated faster and graft survival was poorer in the TRAS group when compared with the control group without TRAS over a mid-term period.

## Supplementary Material

Supplemental Material
