# Supplementary material for: Graft survival after percutaneous transluminal renal stenting for transplant renal artery stenosis (TRAS) is worse compared to matched cadaveric grafts without TRAS
Source: Ren Fail. 2024 Jul 31;46(2):2378211. doi: 10.1080/0886022X.2024.2378211 (PMC11293260; doi:10.1080/0886022X.2024.2378211)
Supplement: Supplemental Material [file IRNF_A_2378211_SM9023.docx]

**Supplementary table 1**

The outcomes of paired patients ( grafts from the same donor) with TRAS

| Parameter | SP group | BA group | NT group |
| --- | --- | --- | --- |
| **No. of patients** | 19 | 2 | 1 |
| Normal serum creatinine | 5 | 1 | 0 |
| Abnormal serum creatinine | 8 | 0 | 0 |
| Died with functional graft | 4 | 0 | 0 |
| Graft loss | 2 | 1 | 1 |
| **Cause of graft loss** |  |  |  |
| Chronic allograft nephropathy | 2 | 1 | 0 |
| Graft kidney artery thrombosis | 0 | 0 | 1 |
| **Outcome after graft failure and return to hemodialysis** |  |  |  |
| Alive on hemodialysis | 2 | 1 | 0 |
| Second transplant and alive at last follow-up | 0 | 0 | 1 |
| **Cause of death** |  |  |  |
| Infection | 4 | 0 | 0 |

SP, stent placement, BA, balloon angioplasty, NT, no treatments;
